# Creasote as an Internal Antiseptic

**Published:** 1898-03-05

**Authors:** 


### CREASOTE AS AN INTERNAL ANTISEPTIC.

Some people talk about giving antiseptics for the cure of internal microbic diseases, and others sneer at them, bringing all kinds of pathological data to show how snugly ensconced the microbes are in living tissues, and how impossible it is for the antiseptics to get at them. Certainly they generally fail, but perhaps they do not give enough. As is well known, Dr. Vivian Poore, when he gives garlic in phthisis, likes his patients to smell of the drug; and Dr. Harry Campbell, writing lately on the tolerance of creasote, says that by giving that drug with cod liver oil, and gradually increasing the amount, he has found little difficulty in getting patients to tolerate drachm doses three or four times a day. When this quantity is given the patient becomes so impregnated with it that all his secretions, and especially the expectoration, acquire a strong odour of the drug. A drachm of creasote three or four times a day is something measurable and substantial, something very different from inhaling a little creasote and eucalyptol.

